# Supplementary material for: Identification of Genes Associated with Gut Microbiota Metabolites in Type 2 Diabetes Mellitus Complicated by Acute Cerebral Infarction Based on Bioinformatics and Mendelian Randomization
Source: Biomedicines. 2026 Oct 17;14(10):2371. doi: 10.3390/biomedicines14102371 (PMC13702449; doi:10.3390/biomedicines14102371)
Supplement: Supplementary file 1 [file biomedicines-14-02371-s001.zip › Figure S5/alpha_estimator_boxplot_goods_coverage.pdf]

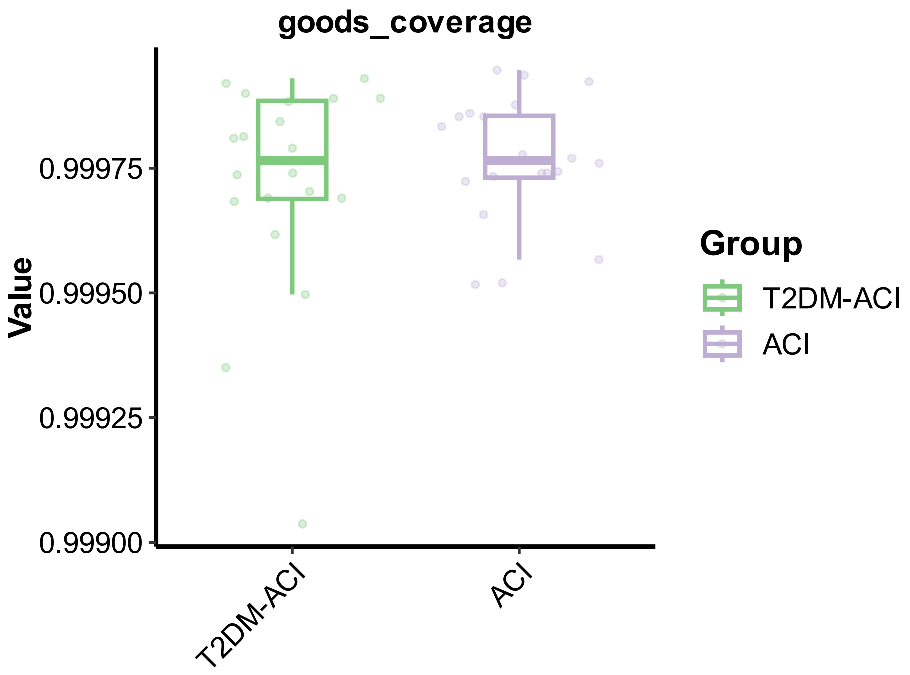
goods_coverage
Value
0.99975
0.99950
0.99925
0.99900
T2DM-ACI
ACI
Group
T2DM-ACI
ACI
